# Engineering Properties of New Claw Connectors for Alkali-Resistant Glass-Fiber-Reinforced Plastics

**DOI:** 10.3390/ma15072631

**Published:** 2022-04-02

**Authors:** Qingbiao Wang, Xu Zhang, Dongya Jing, Zhongjing Hu, Yuanyuan Tian, Dong Wang, Wenxia Liu, Chenglin Tian, Zhenyue Shi, Keyong Wang

**Affiliations:** 1 College of Resources, Shandong University of Science and Technology, Tai’an 271019, China; skd990748@sdust.edu.cn (Q.W.); 150110005@sdust.edu.cn (C.T.); skd996607@sdust.edu.cn (K.W.); 2 College of Safety and Environmental Engineering (College of Safety and Emergency Managemen), Shandong University of Science and Technology, Qingdao 266590, China; 3 National Engineering Laboratory for Coalmine Backfilling Mining, Shandong University of Science and Technology, Tai’an 271019, China; 4 College of Civil Engineering and Architecture, Shandong University of Science and Technology, Qingdao 266590, China; 201982040021@sdust.edu.cn (X.Z.); huyang@sdust.edu.cn (Z.H.); 201982040032@sdust.edu.cn (Y.T.); 201982040033@sdust.edu.cn (D.W.); 5 Zhejiang Xiangsheng Construction Engineering Co., Zhuji 311800, China; jingdy@xsjt.cn; 6 Mountain Tai Composite Industry Research Institute, Tai’an 271019, China; liuwenxia@163.com

**Keywords:** claw connectors, durability, FGFRP, shear resistance, tensile strength

## Abstract

To optimize the engineering properties of connectors, a new claw-shaped alkali-resistant glass-fiber-composite-reinforced connection member was designed in this study. Tensile, shear, and durability tests were conducted on the joint. Moreover, numerical analysis was performed, and the performance of the proposed connector was verified in engineering applications. Hence, the following conclusions hold: (1) At the same shear diameter and anchorage depth, the anchorage performance and shear resistance of claw connectors are better than those of rod connectors. (2) Claw connectors with an anchorage depth of 3.5 cm and a hollow joint with an outer diameter of 14 mm exhibit an excellent overall performance. (3) Alkali-resistant glass-fiber-reinforced plastics exhibit good durability. (4) The ANSYS numerical model can be used to accurately predict the load–displacement variation law of the pull-out and shear of the connectors. (5) Through research, it has been proven that claw-shaped connectors have good pull-out resistance, shear resistance, and durability, and the structure has good stability in engineering applications. Therefore, the structure can provide a significant reference for similar projects.

## 1. Introduction

Assembled buildings are representative of modern industrialized production methods that are being vigorously promoted and applied in China because of their advantages of safe construction, high efficiency, high quality, energy savings, and environmental protection. Connections are important tools in the installation of assembled buildings and are the core components that ensure the reliability of the assembled-building connections. Insulated concrete sandwich-wall-panel (ICSWP) connectors are an integral part of assembled-building connectors; hence, mastering their engineering characteristics is key to ensuring the quality of insulated sandwich-wall-panel installations.

Domestic and foreign researchers have conducted several studies on the structural materials of connectors. Zhang et al. [1] studied the mechanical properties of stainless-steel joist connectors and demonstrated their shear resistance. Kang et al. [2] studied and calculated the thermal-bridging coefficients of steel, aluminum, and stainless-steel material joints, and the results showed that the metallic-material joints did not meet the thermal-insulation criteria in most cases. Greg et al. [3] confirmed the excellent overall performance of fiber-reinforced-plastic (FRP) connections, which was superior to that of conventional steel and polymer materials. Jiang et al. [4] evaluated the shear performance of steel-fiber-reinforced polymeric-material joints. Zhihua et al. [5] proposed an empirical formula for predicting the shear strength of glass-fiber-reinforced-plastic (GFRP) open joints through experimental and finite-element parametric analyses. Clay et al. [6] studied the mechanical properties of different materials, such as glass-fiber-composite reinforcement, stainless steel, galvanized steel, basalt-fiber cloth, and carbon-fiber cloth, to quantify their failure modes and shear resistance. The results indicated that the performance of joints made of FRP materials was outstanding. Prabhu et al. [7] analyzed the tensile and interlaminar shear properties of hybrid Agave sisalana and glass fiber-reinforced polyester composites, and Zhizhou et al. [8] proposed a new I-shaped FRP-material connector for insulated, precast, concrete sandwich-wall panels (IPCSWPs) and performed quasi-static in-plane shear tests to measure the performance of the connector. Pan et al. [9] proposed a C-shaped GFRP connector and quantified its performance under different loading conditions using shear, tensile, and compression tests to derive equations to predict the damage pattern of the connector system. He et al. [10] designed a new type of GFRP I-shaped joint, proposed a new anchorage form with the insertion of anchor reinforcement at the end, and established a punching- and cutting-damage surface-determination method and bearing-capacity formula. Zheng et al. [11] studied the engineering characteristics of rod-shaped, plate-shaped, L-shaped, H-shaped, and slotted joints, and the results indicated that slotted joints are more suitable as internal joints of insulated sandwich walls. Qing et al. [12] proposed a cross-shaped GFRP-material tie member and investigated the effects of its cross-sectional area and arrangement on the engineering properties. Huang [13] conducted shear tests on three types of GFRP connectors, namely, flat plates, corrugated plates, and hexagonal tubes, to re-evaluate the performance of the connectors. Zhang et al. [14] studied the compressive properties of concrete filled with different GFRP tubes, and Eskenati et al. [15] carried out experimental and numerical studies on the mechanical properties of GFRP-type-I connectors.

To examine the performance of connectors, researchers have also conducted extensive research using several research methods. Garai et al. [16,17] proposed a microstructure–mechanical research method for glass composites. Jiang et al. [18] developed a finite-element model using ABAQUS and used the variable-parameter-analysis method to analyze the combined properties of GFRP-material ties. Insub et al. [19] experimentally investigated the fatigue resistance of ICSWPs, and the results showed that fatigue loading significantly affected the load-carrying capacity of the joints. Shao et al. [20] constructed an evaluation system for the pulling parts of ICSWPs using hierarchical analysis and verified the feasibility of the evaluation model. Shi et al. [21] studied the mechanical properties of alkali-resistant glass fiber and concrete composite structures through experiments. Huang et al. [22] conducted static-bending tests and concluded that the stiffness combination of sandwich plates has a tendency to grow nonlinearly with the increase in the shear stiffness of GFRP shear connectors. Won-Hee et al. [23] developed a design model for GFRP mesh sandwich-wall panels to resist bending damage by considering the ultimate and normal-use states. Hou et al. [24] investigated the flexural performance of four types of GFRP connectors: diagonal, round-hole, slotted-hole, and solid webs. The results showed that dense-web GFRP connectors had a high ultimate flexural strength. Lochan et al. [25] proposed a method to investigate the tensile strength of GFRP materials by bending experiments and obtained a correlation between the fracture modulus and tensile strength of GFRP bars. Wu et al. [26] studied the effects of different parameters on the bonding of GFRP materials with concrete. Wu et al. [27] studied the long-term durability of GFRP materials in different environments and established a tensile-strength-prediction model for GFRP bars.

Thus, a large number of studies have been conducted on different materials and structures of connectors and their engineering properties, which provide methodological references and ideas for this study. However, materials with superior performance are not usually adopted, research on multi-directional shear connectors is still not abundant, and some connectors still have the problems of insufficient anchorage force and a complicated prefabricated construction process. Therefore, it is necessary to investigate the engineering properties of novel structural joints made of alkali-resistant glass-fiber materials (FGFRPs).

To solve the problems of complicated prefabricated construction, insufficient anchoring force, and durability of existing connectors, a new claw connector made of FGFRP is designed in this paper based on rod connectors. Although the mechanical properties of the rod connector in the shear direction are isotropic, this is not required in the prefabrication process of the insulated sandwich-wall plate-composite structure of the connector, which simplifies the construction complexity. There are four extended claws at the anchorage ends of the connector, which improve its anchorage strength. Additionally, the connector is made of FGFRP, which significantly improves the overall durability.

The engineering characteristics of the connection were investigated, and numerical simulations were performed using ANSYS finite-element software to analyze the stress–strain of the combined structure of the joints under different stress conditions as well as the failure modes of the structure. The products were verified and applied in the Shennong Zhigu-Time Cloud project in Tai’an City, China, and it had a good performance, thereby providing an important reference for similar projects.

## 2. Materials and Methods

### 2.1. Experimental Materials and Equipment

Based on the spliceable claw connector designed by our team [28], the integrity of the connector was improved by designing the connector rod and anchor ends as a single unit. The anchor ends of the connection ends were claw-type, and they were divided into four extended claws that were evenly distributed in the four directions of the anchor ends. The principal reinforcement was set as a cylinder to avoid the cross-section loss caused by the bar connectors and to improve the anchorage performance.

**Figure 1:**
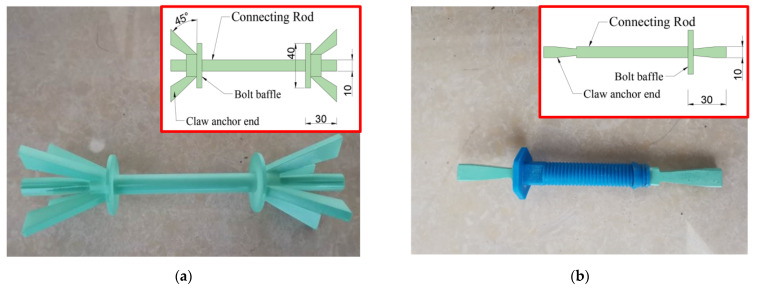
Physical image and dimensions of the connection parts (Unit: mm): (**a**) Claw connectors; (**b**) Rod connectors.

**Figure 2:**
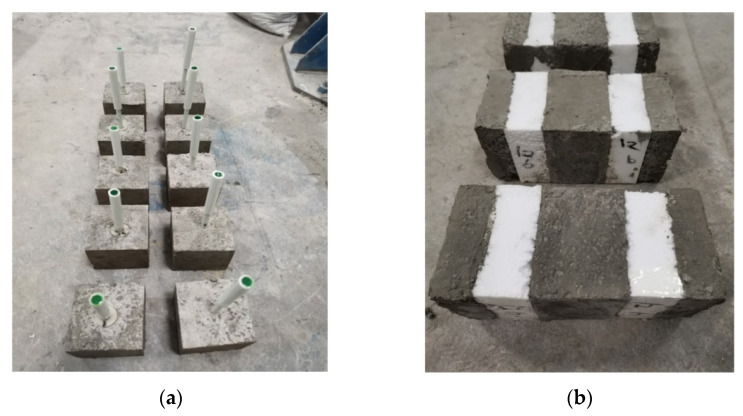
Experimental apparatus and test pieces: (**a**) Pull-out specimen; (**b**) Shear specimen.

The new connector material was made of FGFRP, which was developed by Shandong SFT Industrial Co., Ltd. The samples were processed using the company’s production line (Figure 1). Because rod connectors are widely used in engineering, rod connectors were also produced using FGFRP and processed (Figure 2) for use in comparative mechanical-property experiments. The mechanical properties of FGFRP are listed in Table 1.

The parameters of the main equipment used in the experiments are listed in Table 2.

Cement, sand, gravel, water, and other materials were also used in the experiments.

### 2.2. Experimental Protocol

#### 2.2.1. Pull-Out Test

Based on the requirements of the Fiber Reinforced Plastic Connectors for Prefabricated Insulated Walls JG/561-2019 [29], the cross-sectional area of the connector should not be less than 50 mm^2^. According to a study by Woltman et al. on the mechanical properties of GFRP connectors with diameters ranging from 6 to 13 mm [3], the connecting rod was designed as a solid cylinder with a diameter of 10 mm for the pull-out experiments.

Relevant studies have proven that when the thickness of the outer-leaf wall panel exceeds 80 mm, the bearing capacity of the insulated sandwich-wall panel gradually decreases [18].

In summary, six sets of experiments were designed for different anchorage depths to investigate the tensile performance of joints at different anchorage depths. The anchorage depths varied along the length of the connecting rod, whereas the dimensions of the claw-type anchorage end remained the same. The designed strength grade of the concrete was C40, and the experimental specimen was a square of 150 × 150 × 150 mm. Because the length of the FGFRP material was slightly increased, the displacement due to extension was not considered in the experiments. The specimen parameters of the pull-out-test specimen are listed in Table 3, where T0 is the bar connection and T1–T5 are the claw connections. A standard test block of dimension 150 × 150 × 150 mm was made from the same batch of concrete to test the compressive strength of concrete.

**Figure 3:**
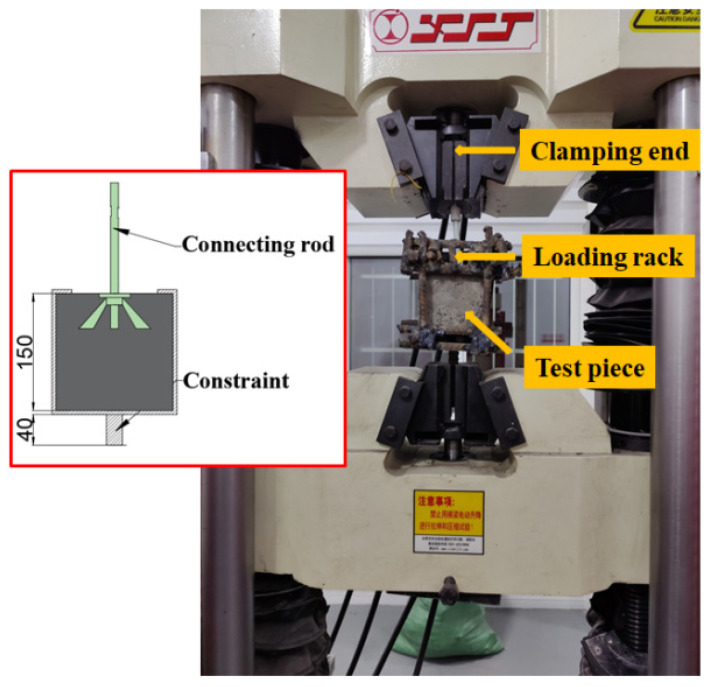
Schematic diagram of the pull-out-specimen structure (Unit: mm).

During the experiment, the lower part of the loading frame was fixed to the clamping end of a universal-testing machine, and the upper part of the loading frame was used to fix the specimen, as shown in Figure 3. The universal-testing machine was used for uniform loading along the vertical direction at a loading speed of 1 kN/min [29]. The loading was increased until the specimen was pulled out or produced structural damage, and pertinent data were recorded.

#### 2.2.2. Shear Test

**Figure 4:**
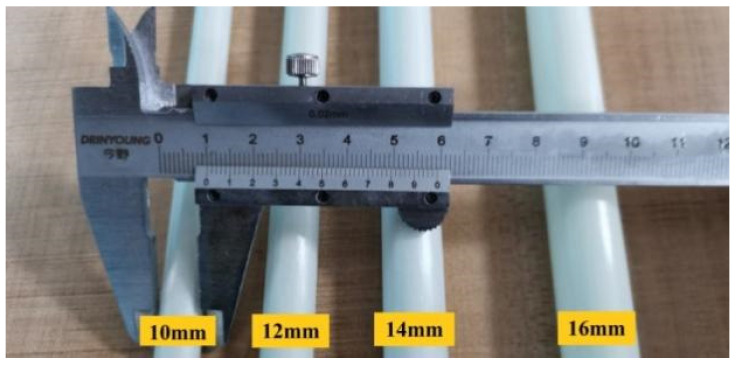
Hollow connecting rods with different outer diameters (Unit: mm).

To test the effect of the cross-sectional parameters on the shear characteristics of the new connectors and minimize thermal-bridging losses [2], hollow connectors with the same cross-sectional area but different inner and outer diameters were designed, where L0 is the bar connection and L1–L5 are the claw connections. The shear-specimen parameters are listed in Table 4. The main reinforcements of the remaining bars were hollow cylinders of various sizes, as shown in Figure 4.

**Figure 5:**
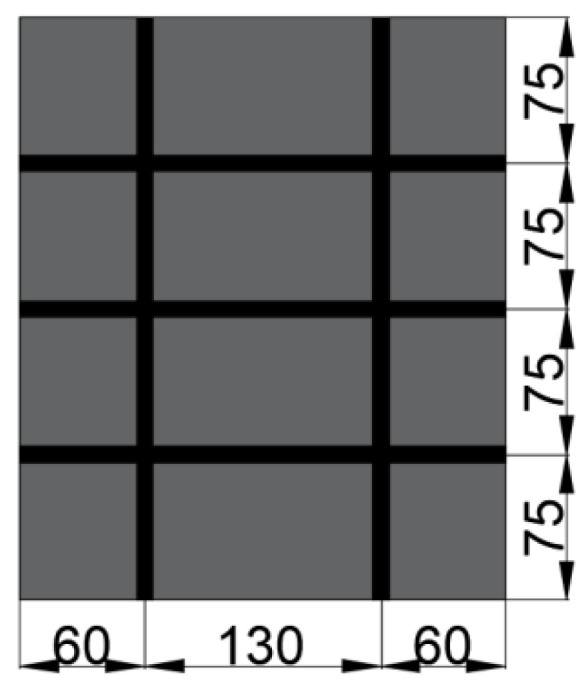
Inner- and outer-leaf wall-panel-reinforcement diagram (Unit: mm).

The reinforcement material for the inner- and outer-leaf wall plates of the shear specimens was a galvanized high carbon–steel reinforcement material of diameter 2 mm, and the reinforcement rate was set as 0.2% [30], as shown in Figure 5.

**Figure 6:**
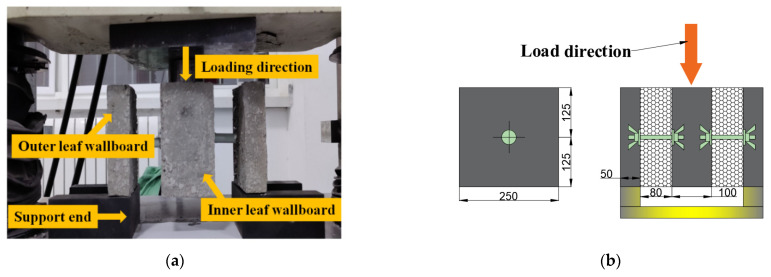
Schematic diagram of shear specimen and loading method (Unit: mm): (**a**) Specimen-loading diagram; (**b**) Specimen size.

Meanwhile, the anchoring depth and thickness of the protective layer were not less than 30 and 25 mm, respectively, and the inner-leaf wall panel was twice the thickness of the outer-leaf wall panel. Figure 6 shows the loading method, in which the lower side of the outer-leaf wall plate was fixed and the inner-leaf wall plate was loaded at a uniform rate of 1 kN/min using a universal-testing machine [29]. The load was applied until either the displacement of the specimen exceeded 1 cm or shear damage occurred.

#### 2.2.3. Durability Test

A connector with a diameter of 10 mm and an anchorage depth of 30 mm was selected for the alkali-solution-immersion test. The alkali solution was formulated as shown in Table 5 [29]. The connectors soaked in the alkali solution were made into pull-out and shear specimens in order to conduct the pull-out and shear-strength tests. The test values were then compared with the experimental values.

The connections were placed in a constant-temperature-and-humidity-curing machine with the solution temperature controlled at 60 ± 3 °C. The alkali solution was measured and periodically adjusted during the experiment to ensure a stable PH value [31]. The durability-test specimens were divided into four groups, each group containing five pull-out- and five shear-test specimens. The soaking periods of the four groups were 30, 60, 90, and 180 days.

## 3. Analysis of Experimental Results

The strength test was conducted on standard specimens maintained under the same conditions before the experiment, and the average compressive strength of the specimens was measured as 43.22 MPa, which satisfied the design requirements.

### 3.1. Analysis of Pull-Out Test Results

**Figure 7:**
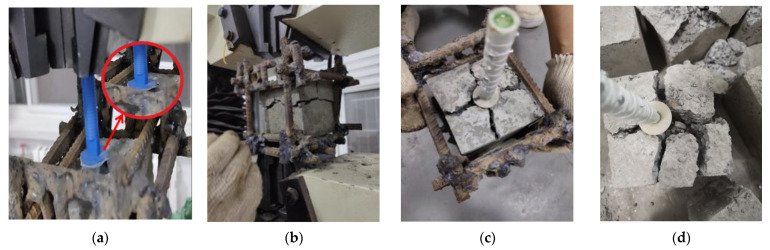
Damage state of pull-out-test specimens: (**a**) Rod connectors are pulled out; (**b**) Lateral concrete fracture of the claw connectors; (**c**) Cross-splitting of the upper part of the claw connectors; (**d**) Broken anchor-end jaws.

As shown in Figure 7, in the pull-out test, the rod connectors were directly pulled out, and the concrete around them produced few cracks. Additionally, the rod connectors were not damaged; however, the concrete of the claw-connector specimens produced cross-shaped splits along the direction of the claw and penetration fractures in the concrete at the bottom of the anchored end. The concrete damage state was similar to the phenomenon observed when the anchorage depth was increased. At anchoring depths of 4, 4.5, and 5 cm, the jaws of the connector were fractured, and the connector rod did not produce damage.

In summary, claw connectors have a larger anchorage range and stronger anchorage than rod connectors. The experimental phenomenon showed that the anchoring end of claw connectors improved the anchoring performance of the claw when the anchoring depths were 3 and 3.5 cm. As the anchorage depth continued to increase, the jaws were fractured, resulting in a mechanical loss of material.

As listed in Table 6, the average ultimate tensile strength of the claw connectors was higher than that of the rod connectors. Additionally, the average ultimate load of the claw connectors gradually increased with an increase in the anchorage depth.

Data from the pull-out experiments showed that the stress–strain patterns were highly similar for the same group of specimens. In different experimental groups, data with similar ultimate loads and average values were selected, and their load–displacement curves were plotted for comparative analysis.

**Figure 8:**
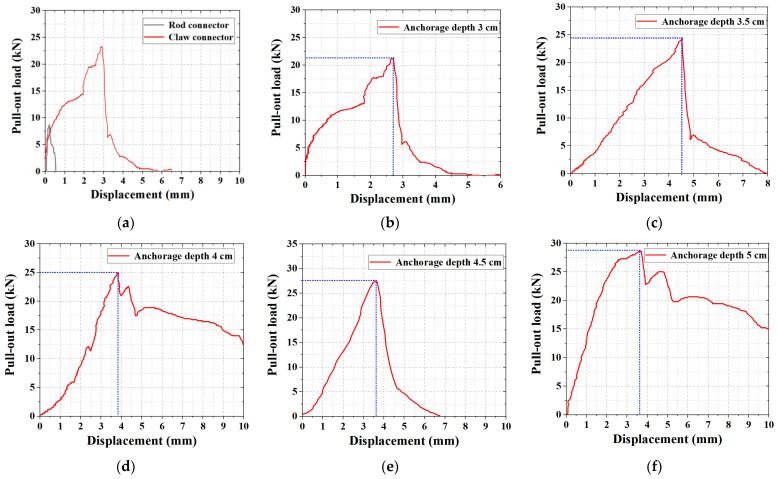
Load–displacement curves of the pull-out test: (**a**) Comparison of the rod and claw connectors. Tensile strengths of the rod and claw connectors at (**b**) 3 cm anchorage depth; (**c**) 3.5 cm anchorage depth; (**d**) 4 cm anchorage depth; (**e**) 4.5 cm anchorage depth; (**f**) 5 cm anchorage depth.

As shown in Figure 8, the load–displacement changes exhibited the same pattern during the pull-out experiment. First, as the displacement increased, the load rapidly increased and reached a peak, after which it rapidly decreased, damaging the specimen.

As shown in Figure 8a, the maximum tensile strength of the rod connectors was 8.75 kN, whereas that of the claw connectors was 21.14 kN, which was 2.42 times that of the rod connectors. At the same anchorage depth, the tensile strength of the claw connectors was higher than that of the rod connectors.

As shown in Figure 8b–f, the ultimate tensile strengths of the claw connectors at anchorage depths of 3–5 cm were 21.14, 24.33, 25.03, 27.48, and 28.53 kN, respectively. As the anchorage depth increased, the tensile strength of the specimens was improved; however, the overall strength and strength base were relatively small, which is similar to the results of a previous study [5]. When the anchorage depth increased from 3 to 3.5 cm, the ultimate tensile strength of the specimens had the largest increase. With the increase in the anchorage depth, the increase rate of the tensile strength of the specimens gradually decreased. This is because the claw at the anchorage end has a 45° inclination angle. During the experiment, the force of the claw decomposed into axial and shear stresses. When the force increased to a certain value, the claw sheared into the concrete. At this time, the connecting rod bore the main pull-out stress. Continuing to increase the anchorage depth has a poor effect on the improvement of the mechanical properties of the overall structure. In addition, an increase in the anchorage depth led to an increase in the thickness and weight of the outer-leaf wall plate, which resulted in higher requirements for the shear resistance of the connector.

In summary, the structure of a joint has a significant influence on its ultimate tensile strength. Although the anchorage depth affects the ultimate tensile strength, the effect is small. Combined with the analysis of the damage state of the claw connectors, the optimal anchorage depth of the claw connectors was approximately 3.5 cm. When the ultimate tensile strength of the joint was four times the standard value, the standard value of the tensile bearing capacity was ≥6.0 kN [29].

**Figure 9:**
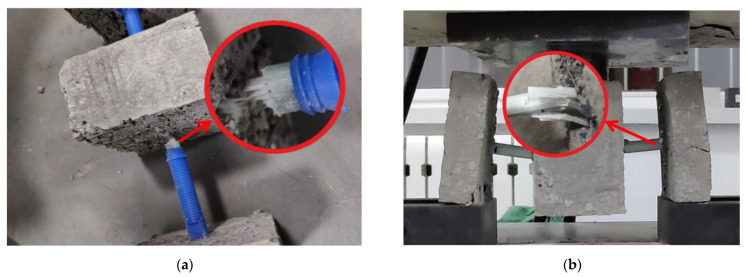
Shear-specimen damage state: (**a**) Damage state of the rod connector; (**b**) Damage state of the claw connector.

As shown in Figure 9, in the shear-strength test, the rod connectors sheared off at the connection with the inner-leaf wall panel, whereas no significant damage was observed at this point in the inner- and outer-leaf wall panels. The claw connectors also failed at the connection with the inner- and outer-leaf wall panels, and when damaged, some of the outer-leaf wall panels were bent, and cracks were evident in the inner-leaf wall panels. Compared to rod connectors, claw connectors have a higher degree of combination with inner- and outer-leaf wall panels.

### 3.2. Analysis of Shear Test Results

As listed in Table 7, the average ultimate shear strength of the claw connectors was higher than that of the rod connectors. At the same cross-sectional area, connecting rods with different inner and outer diameters had similar shear strengths.

The shear experimental data showed that the stress–strain patterns were highly similar for the same group of specimens. In different experimental groups, data with similar ultimate loads and average values were selected, and their load–displacement curves were plotted for comparative analysis.

**Figure 10:**
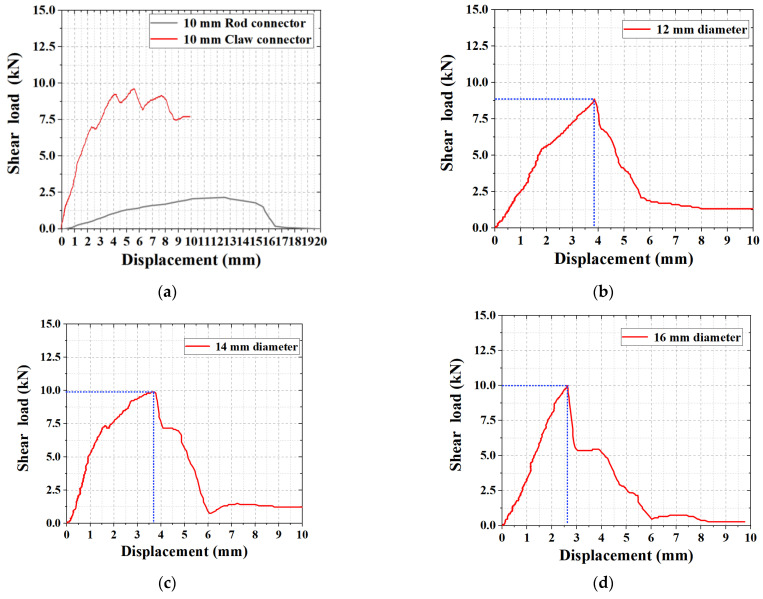
Load–displacement curves of the shear test: (**a**) Comparison of rod and claw connectors. Shear strengths of claw connectors of (**b**) 12 mm outer diameter; (**c**) 14 mm outer diameter; (**d**) 15 mm outer diameter.

As shown in Figure 10, the shear load–displacement curves exhibited a similar pattern. In the early loading stage, the load increased rapidly and reached a certain value before the growth rate reduced, and the load–displacement curve presents the yield form. When the limit value was exceeded, the load began to rapidly decrease, and the curve eventually flattened out.

As shown in Figure 10a, the ultimate shear load of the rod connectors was 2.17 kN, whereas of the claw connectors was 9.72 kN, which was 4.48 times that of the rod connectors for the same joint-rod diameter. The shear resistance of the claw connectors was better than that of the rod connectors for the same connection-rod diameter.

As shown in Figure 10b–d, when the outer diameter of the connecting rod of the claw connector was 10 mm, the ultimate load of the connecting member was 9.72 kN, and the displacement was 6.29 mm. When the outer diameter was 12 mm, the ultimate shear strength of the joint was 8.85 kN, and the displacement was 3.85 mm. When the outer diameter was 14 mm, the ultimate shear strength of the joint was 9.89 kN, and the displacement was 3.72 mm. When the outer diameter was 16 mm, the ultimate shear strength of the joint was 9.98 kN, and the displacement was 2.93 mm. In the overall analysis, the ultimate shear strengths of the four connection diameters were similar. As the diameter increased, the displacement of the specimen with the ultimate load gradually decreased; that is, the stiffness of the connector increased, which was also confirmed in a previous study [32]. When the diameter was increased from 10 to 12 mm, the maximum reduction in ultimate load and displacement of the specimens was 63.38%. Meanwhile, the yielding phase of the shear load–displacement curve became less pronounced when the diameter of the connecting rod increased, and the failure state of the structure was nearly brittle. This is because when the external diameter increases, the internal diameter continues to decrease, and more materials in the structure are used to improve the rigidity of the connecting rod. This failure state is unfavorable for a structure subjected to a fatigue load during use. Based on the performance analysis, the optimal diameter of the claw connectors should be 12 and 14 mm. When the outer diameter is 14 mm, the ultimate shear load of the connector becomes relatively large, which is more advantageous. When the outer diameter of the connection rod was 14 mm, the standard value of the shear bearing capacity was ≥0.9 kN [29], and the shear bearing capacity of the connection was 12.36 times the standard value.

### 3.3. Analysis of Durability Test Results

**Figure 11:**
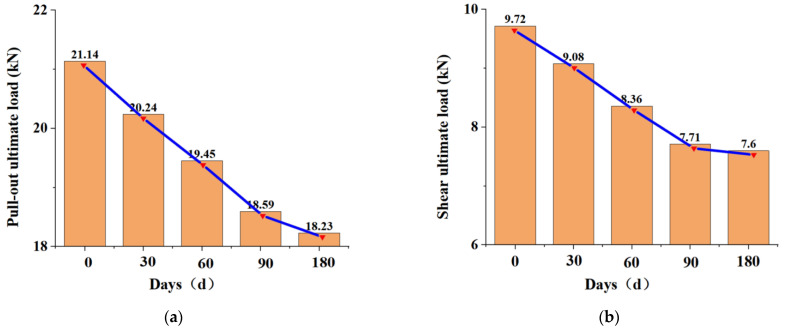
Load–displacement curves of the durability test: (**a**) Load–displacement curve for the pull-out test; (**b**) Load–displacement curve for the shear test.

A durability-test specimen was selected with an anchoring depth of 30 cm and a connecting-rod diameter of 10 mm. Experimental tests were conducted to obtain the residual strength of the connections on different days, as shown in Figure 11.

Through the calculation of data in Figure 11a, it can be obtained that the average values of residual tensile strength of the claw connectors were 95.74%, 92.01%, 87.96%, and 86.24% of the normal values after 30, 60, 90, and 180 days of immersion in the alkali solution, respectively. It was observed from the line graph that as the days increased, the rate of decrease in the ultimate tensile strength of the joint tended to level off. When subjected to a pull-out load, the connecting rod did not reach the ultimate tensile strength, and the drop in the load–displacement curve at this time was caused by the decrease in the shear force of the anchored end jaws.

Through the calculation of data in Figure 11b, the average values of the residual shear strength of the claw connectors were 93.42%, 86.01%, 79.31%, and 78.22% of the normal values after 30, 60, 90, and 180 days of immersion in the alkali solution, respectively. The rate of decrease in the ultimate shear strength of the connectors tended to level off with increasing time. Under immersion in the alkali solution, the glass fibers and matrix in the connecting rods were damaged, and the shear strength decreased.

In summary, after 180 days of immersion, the residual tensile strength of the joints was 86.24%, whereas the residual shear strength was 78.22%, which satisfied the requirements that the residual strength should be greater than 70% [29] and have a relatively rich safety factor.

## 4. Numerical Simulation

**Figure 12:**
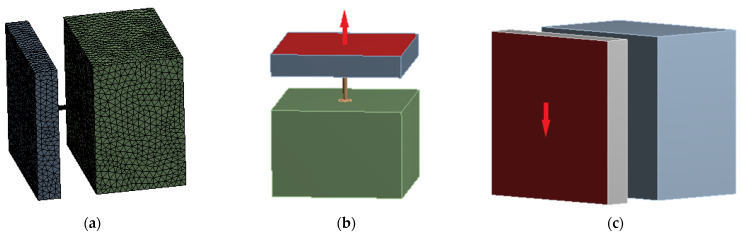
Precast, insulated, concrete sandwich-wall-panel unit-force model: (**a**) Grid division; (**b**) Pull-out-force loading direction; (**c**) Shear-force loading direction.

A numerical model was constructed for the new connector described in Section 2. The optimal anchorage depth and external diameter of the connector were 3.5 cm and 14 mm, respectively. To further analyze the mechanical properties of the optimal connector structure, the external diameter of the connector was 14 mm, and the anchorage depth was 3.5 cm in the numerical model. To simulate the force characteristics of the model, an insulated wall panel was considered as the object of study. Constraints were applied to the inner-leaf wall panels of the insulated wall, and different sizes of concentrated loads were applied to the outer-leaf wall panels during the simulation with vertical and horizontal load directions. The theoretical analysis showed that applying a unilateral shear force to the outer-leaf wall panel had the same shear effect as the experimental loading. Figure 12 shows the details of the model.

LINK8 units were used for the connection and SOLID65 units were used for the concrete. The Poisson’s ratio V of the concrete was chosen to be 0.3, and the values of the remaining parameters are listed in Table 8. In the finite-element analysis, the function of the large deformation of concrete was turned on. The Poisson ratio V of the FGFRP material was considered as 0.38, and the rest of the parameters are listed in Table 1.

Ft is the tensile strength, Fc is the compressive strength, ψ is the expansion angle, *f_b0_/f_c0_* is the ratio of the biaxial ultimate compressive strength to the uniaxial compressive ultimate strength, *K_c_* is the ratio of the second stress invariance on the tensile radial plane to that on the compressive radial plane, and *µ* is the viscosity coefficient.

### 4.1. Comparative Analysis of the Experimental and Simulated Pull-Out Results

**Figure 13:**
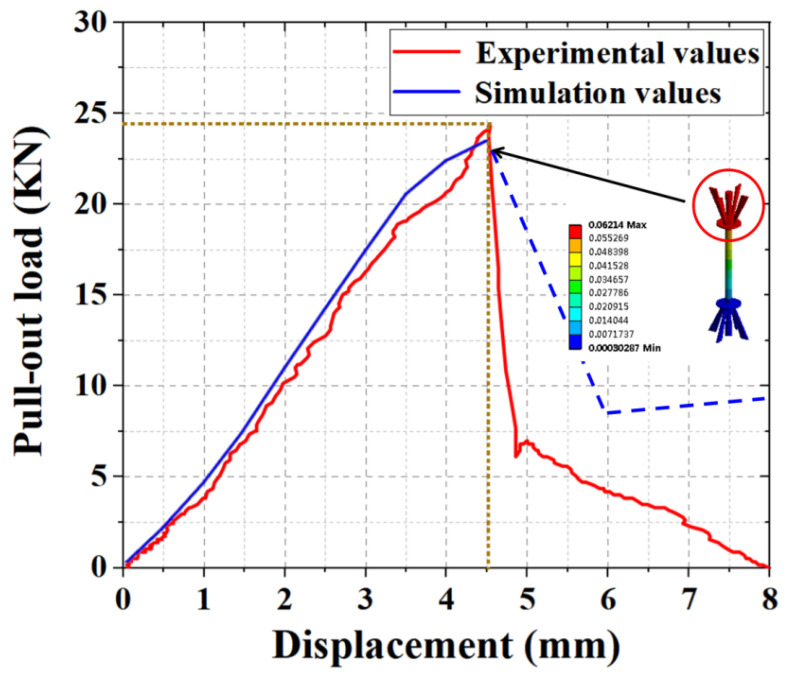
Comparison load–displacement curves of the experimental and simulated pull-out tests.

As shown in Figure 13, the pull-out simulation curve had the same variation pattern as the experimental curve at the preloading period and had a good fit. The experimental ultimate tensile strength was 24.33 kN, whereas the experimental simulated tensile strength was 23.13 kN, and the simulated value was 95.10% of the experimental value, with a deviation of 4.9%. In a similar study, Huang et al. [13] compared the numerical simulation results with the experimental results, and the average Pf and limit Kf deviations were 6.0% and 8.5%, respectively. Furthermore, the data in this study showed that the simulated ultimate load value was slightly smaller than that of the experimental value because the small loading range increased owing to the weight of the experimental specimen during the experiment, and the viscosity coefficient of concrete changed over a small range owing to the influence of humidity during the experiment. However, the model accurately predicted the experimental results in the load-increasing stage. As shown by the strain cloud diagram, the model exhibited the largest deformation at the anchorage position of the tensile end and was prone to damage. This was confirmed by the fracture of the anchor jaws during the experiment, which proved the correctness of the experimental results and the rationality of the numerical simulation.

### 4.2. Comparative Analysis of the Experimental and Simulated Shear Results

**Figure 14:**
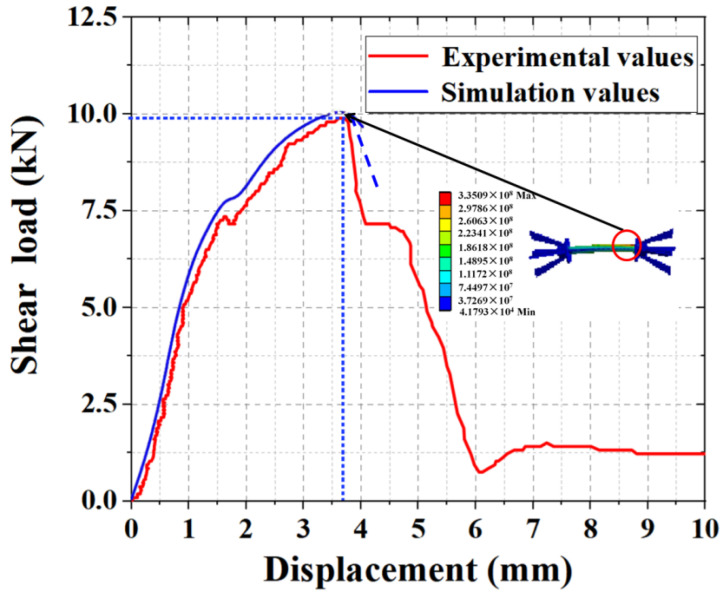
Shear experiment and simulated load–displacement comparison curve.

As shown in Figure 14, in the preloading period, the shear simulation curve had the same variation pattern as the experimental curve, and the load increased sharply with an increase in displacement. When the load reached a certain value, the load–displacement curve appeared similar to the yield phenomenon and began to drop sharply when the load exceeded the limit value. The ultimate load of shear simulation was 10.14 kN, whereas the experimental ultimate value was 9.89 kN. The simulated value was 102.50% of the experimental value, with a deviation of 2.50%. In a similar study, Zhizhou et al. [8] compared the numerical-simulation results with the experimental results and analyzed the reasons for the differences. The data in the study showed that the simulated ultimate load was slightly larger than the experimental value because the numerical simulation was completely idealized, and the concrete was affected by humidity, the displacement of the reinforcement position, and lack of vibration density during the experiment, resulting in a deviation between the experimental ultimate load and the simulation value. However, the model accurately predicted the experimental results in the load-increasing stage. As shown in the strain cloud diagram, the maximum strain was generated at the interface between the outer-leaf wall plate and connecting rod, which was consistent with the experimental damage results. Hence, the accuracy of the experimental results and reasonableness of the numerical simulation were confirmed.

## 5. Engineering Applications

### 5.1. Project Overview

The project is located in the new construction area of Tai’an City, Shandong Province, China, directly opposite the eastern gate of Shandong First Medical University. It is bordered on the west side by the Great Wall Road, on the south side by the East Tianmen Street, and on the east and north sides by municipal planning roads. The site has an overall “mouth” shape, and the original topography of the site is high in the south and low in the north. The longest distances from east to west and north to south are approximately 232 and 223 m, respectively. The planned construction land area of the project is about 40,000 m^2^, and the total construction area of the project is about 120,000 m^2^.

### 5.2. Monitoring Program

**Figure 15:**
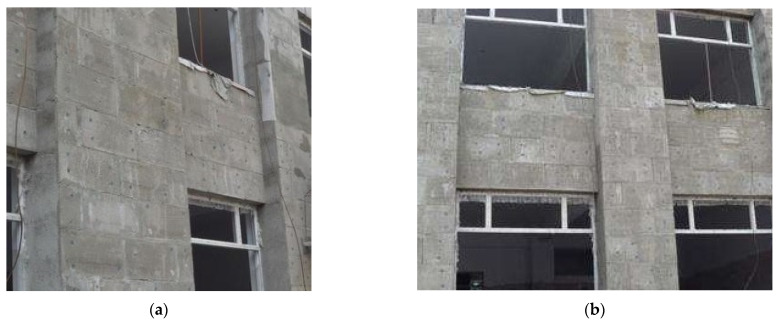
Building #1 exterior insulated wall panel: (**a**) Side view; (**b**) Front view.

Building #1was designed as a frame structure, and the foundation form is independent. The number of building floors is two floors above the ground, and the construction area is 1194.68 m^2^. The building insulation exterior wall adopts the claw-type connector insulated concrete sandwich-wall-panel structure designed in this study. The anchorage depth of the claw-type connector is 35 mm, the thickness of the insulation layer is 60 mm, and the size of the exterior-leaf wall panel is 800 × 400 × 60 mm, as shown in Figure 15.

**Figure 16:**
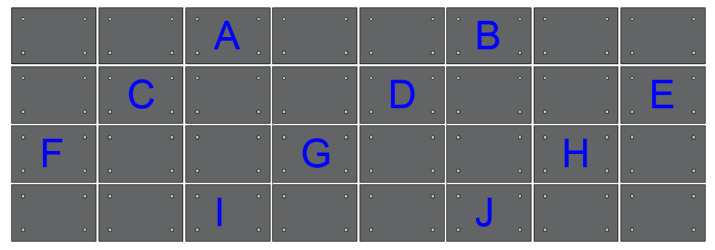
Layout map of the regional monitoring points.

To verify the engineering application performance of the new connectors, the exterior insulated wall panel of building #1 was selected as the monitoring object, and four connectors were set up on the wall panel with monitoring points. The shear displacement was monitored using prebuilt strain gauges [33], and the relative displacement of the monitored wall panels was considered as the average of the four connections. The monitoring numbers are A–J, and the layout of the monitoring points are shown in Figure 16.

### 5.3. Analysis of the Monitoring Results

**Figure 17:**
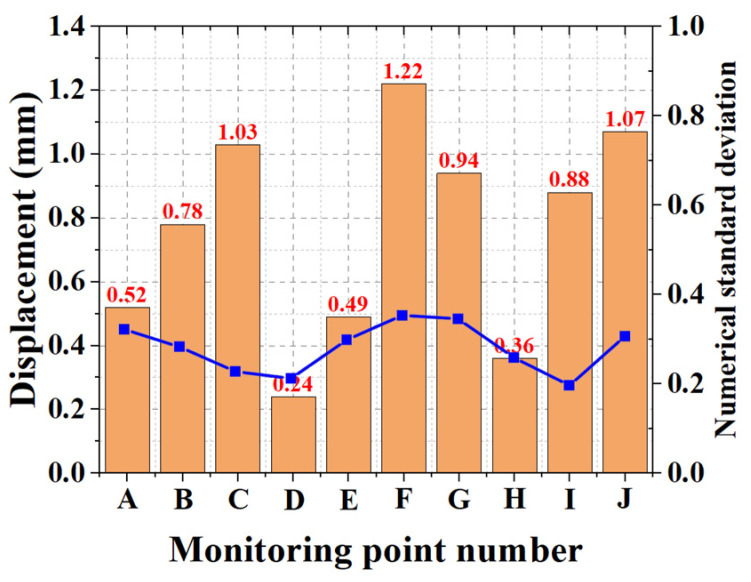
Monitoring-point displacement.

In Figure 17, the histogram showed the displacement of each monitored connection bar, and the value was the average value of the four connection bars within the same insulated wall panel. The results indicated that the maximum displacement was 1.22 mm, which did not exceed the standard value of 254 mm [34]. The line graph shows the standard deviation of each mean displacement value, where the maximum value of the standard deviation was 0.37, indicating that the connection material had strong overall stability. In summary, the practical application of the connection meets the design requirements and has a relatively good safety reserve.

## 6. Discussion

In this study, a claw-type connector was designed, which showed good performance in terms of pull-out resistance, shear resistance, and durability. Although the performance of the proposed connector meets the requirements of engineering applications, in practical applications, the damage arising from long-term environmental effects on the external thermal insulation wall structure is becoming increasingly prominent. For example, the structural vibration caused by wind load and the influence of high and low temperatures on the material properties are issues that need further consideration for the long-term reliability of new structures. Based on the research of the material structure and basic performance, experimental and theoretical studies need to be conducted to investigate the influence of environmental factors on the performance of connectors in order to improve the structural-design level of connectors and enhance their ability to resist force majeure.

## 7. Conclusions

The following conclusions were drawn based on this study:

(1) In the comparison experiment with the same anchorage depth and insulation thickness, the average pull-out and shear loads of the claw-type connector were 21.27 and 9.69 kN, respectively. Similarly, the average pull-out and shear loads of the rod connector were 8.70 and 2.21 kN, respectively, indicating that the anchorage and shear performances of the claw connector are better than those of the rod connector.
(2) In the experiments, it was determined that the optimal cross-section parameters of the hollow connecting rod were 14 mm in outer diameter and 9.8 mm in inner diameter, and the optimal anchorage depth of the anchorage end was 3.5 cm. Moreover, the ultimate tensile strength was 24.33 kN, which was four times the standard value, whereas the shear capacity was 9.89 kN, which was 12.36 times the standard value.
(3) After the durability test, the residual tensile strength of the claw connectors was 86.24% of the normal value, whereas the residual shear strength was 78.22% of the normal value after 180 days of alkali corrosion. This indicates that the FGFRP material had superior durability.
(4) A numerical model of the insulation wall board was constructed using ANSYS finite-element-analysis software. The simulation results showed that the simulated pull-out and shear load–displacement curves had similar variations with those of the experimental load–displacement curves, with the deviation of the pull-out and shear limit loads less than 5%. This indicates that the numerical model can accurately predict the load–displacement relationship of the connector.
(5) In the engineering application, the maximum shear displacement of the claw-type connector was 1.22 mm, and the maximum standard deviation of the displacement of the same insulation board was 0.37. Therefore, the performance of the connector is stable and has a high engineering application value.

## Figures and Tables

**Table 1:** Mechanical properties of the material.

Material	Tensile Strength (fy/MPa)	Shear Strength (fv/MPa)	Modulus of Elasticity (Es/10^5^ MPa)	Elongation
FGFRP	1086	281	0.54	2%

**Table 2:** Main experimental equipment parameters.

Equipment	Specification Model	Precision	Usage
Standard constant-temperature-and-humidity-maintenance machine	40	/	Specimen maintenance
Electronic universal-testing machine	WAW-600F	0.5%	Strength-resistance test

**Table 3:** Parameters of the pull-out-test specimen.

Group	Number	Insulation-Layer Thickness (mm)	Diameter of Connecting Rod (mm)	Length of Connecting Rod (mm)	Anchorage Depth (mm)
T0	T0/1–T0/5	80	10 × 6 (Board type)	140	30
T1	T1/1–T1/5	80	10	140	30
T2	T2/1–T2/5	80	10	150	35
T3	T3/1–T3/5	80	10	160	40
T4	T4/1–T4/5	80	10	170	45
T5	T5/1–T5/5	80	10	180	50

**Table 4:** Shear test specimen parameters.

Group	Number	Insulation-Layer Thickness (mm)	Outer Diameter of Connection (mm)	Inner Diameter of Connection (mm)	Anchorage Depth (mm)
L0	L0/1–L0/5	80	10 × 6 (Board type)		30
L1	L1/1–L1/5	80	10	0	30
L2	L2/1–L2/5	80	12	6.6	30
L3	L3/1–L3/5	80	14	9.8	30
L4	L4/1–L4/5	80	16	12.5	30

**Table 5:** Alkali-solution proportion.

Solution	Grams of Solute in 1 L of Water (g/L)		
	Ca(OH)_2_	KOH	NaOH
Alkali solution	118.5	4.2	0.9

**Table 6:** Experimental data of the tensile strength of connections.

Group	Ultimate Pulling Load (kN)					Average Load (kN)	Standard Deviation
T0	T0/1	T0/2	T0/3	T0/4	T0/5	8.70	0.12
	8.34	8.75	8.25	9.02	9.13		
T1	T1/1	T1/2	T1/3	T1/4	T1/5	21.27	3.70
	21.14	23.58	20.37	18.22	23.02		
T2	T2/1	T2/2	T2/3	T2/4	T2/5	24.28	1.63
	22.19	24.33	25.09	23.82	25.98		
T3	T3/1	T3/2	T3/3	T3/4	T3/5	25.13	1.77
	24.39	25.87	25.03	27.15	23.21		
T4	T4/1	T4/2	T4/3	T4/4	T4/5	26.81	5.30
	23.04	25.68	28.01	29.83	27.48		
T5	T5/1	T5/2	T5/3	T5/4	T5/5	28.47	1.68
	26.25	29.71	28.06	28.53	29.79		

**Table 7:** Experimental data of the tensile strength of connections.

Group	Ultimate Shear Load (kN)					Average Load (kN)	Standard Deviation
L0	L0/1	L0/2	L0/3	L0/4	L0/5	2.21	0.05
	1.93	2.05	2.17	2.52	2.37		
L1	L1/1	L1/2	L1/3	L1/4	L1/5	9.69	0.27
	9.72	9.51	8.8	9.92	10.44		
L2	L2/1	L2/2	L2/3	L2/4	L2/5	8.82	4.67
	4.68	8.85	9.91	10.83	9.82		
L3	L3/1	L3/2	L3/3	L3/4	L3/5	9.77	1.02
	11.14	10.08	9.72	9.89	8.01		
L4	L4/1	L4/2	L4/3	L4/4	L4/5	9.98	0.48
	10.57	9.99	9.82	9.20	10.34		

**Table 8:** Experimental data of the tensile strength of connections.

Density (kg·m^3^)	Ft (MPa)	Fc (MPa)	Ψ (°)	*f*_b0_/*f*_c0_	*K*_c_	*µ*
2500	2.39	42.5	15°	1.16	0.667	0.0005

## Data Availability

The data presented in this study are available on request from the corresponding author.
